# Impact of Polymorphisms in Casein Kinase 1 Epsilon and Environmental Factors in Oral Cancer Susceptibility

**DOI:** 10.7150/jca.34592

**Published:** 2019-08-28

**Authors:** Shu-Hui Lin, Mu-Kuan Chen, Julia Hueimei Chang, Bharath Kumar Velmurugan, Madhavi Annamanedi, Shih-Chi Su, Kun-Tu Yeh, Shun-Fa Yang

**Affiliations:** 1 Department of Surgical Pathology, Changhua Christian Hospital, Changhua, Taiwan; 2 Department of Medical Laboratory Science and Biotechnology, Central Taiwan University of Science and Technology, Taichung, Taiwan; 3 Department of Otorhinolaryngology-Head and Neck Surgery, Changhua Christian Hospital, Changhua, Taiwan; 4 Department of Biotechnology, Asia University, Taichung, Taiwan; 5 National Institute of Animal Biotechnology, Gachibowli, Hyderabad, India; 6 Whole-Genome Research Core Laboratory of Human Diseases, Chang Gung Memorial Hospital, Keelung, Taiwan; 7 School of Medicine, Chung Shan Medical University, Taichung, Taiwan; 8 Institute of Medicine, Chung Shan Medical University, Taichung, Taiwan; 9 Department of Medical Research, Chung Shan Medical University Hospital, Taichung, Taiwan

**Keywords:** Casein kinase 1 epsilon, oral cancer, polymorphism

## Abstract

In Taiwan, the incidence rate of oral cancer is constantly increasing. Polymorphisms and lifestyle habits are major contributing factors to the development of oral cancer in such cases. Casein kinase 1 epsilon (*CK1ε*) gene expression plays a role in numerous cancers, and the knockdown of *CK1ε* induces tumor cell-selective cytotoxicity. The present study was designed to determine the effects of *CK1ε* gene polymorphisms combined with environmental carcinogens on susceptibility to developing oral squamous cell carcinoma and its clinicopathological status. Four single-nucleotide polymorphisms (SNPs) in *CK1ε* gene (rs135745, rs135764, rs1997644 and rs2075984) from 741 oral cancer patients and 462 healthy controls were analyzed using real-time polymerase chain reaction. Our results shown that variant types (GC) of *CK1ε* polymorphic rs135745 exhibited a significantly higher risk of 1.41 (95% confidence interval [CI]: 1.036-1.919) for oral cancer than did wild type alleles. Furthermore, these *CK1ε* gene SNPs along with betel-quid chewing and/or tobacco use further increased susceptibility to oral cancer. Moreover, variant genotypes (GC+CC) of CK1ε rs135745 were significantly associated with lymph node metastasis. These results suggested that the *CK1ε* gene polymorphism is associated with the clinicopathological development of oral cancer and increases individuals' susceptibility to environmental carcinogens (e.g., smoking and betel-quid chewing) in terms of developing oral cancer.

## Introduction

Oral cancer is estimated to be in the top 10 most common cancers worldwide, and is characterized by poor prognosis and late detection. Since 2003, oral cancer has been the fourth leading cause of cancer deaths among men in Taiwan 1. The occurrence and growth of oral cancer depends on DNA sequence modifications known as single-nucleotide polymorphisms (SNPs). Along with genetic factors, environmental carcinogens associated with betel-quid (areca nut) chewing, tobacco use, and alcohol intake are major suggested causes of oral squamous cell carcinoma in Taiwan 2.

A member of the casein kinase I protein family, the casein kinase 1 epsilon (CK1ε) gene encodes serine/threonine protein kinase. Members of the CK1 gene family (α, β, γ1, γ2, γ3, and δ) have been shown to be involved in the regulation of cytoplasmic and nuclear processes as well as DNA replication and repair. Furthermore, studies have shown that in colon adenocarcinoma cells, human pancreatic cells, and salivary gland cancer cells, CK1ε phosphorylates vital proteins of the Wnt signaling pathway to maintain cell division and tumor growth 3. In our previous studies, we have demonstrated that decreased cytoplasmic CK1ε expression correlates with low survival rates in patients with oral and hepatocellular carcinomas 4, 5. These results have suggested that CK1ε could be utilized as a prognostic predictor for oral and hepatocellular cancers and act as a tumor suppressor agent 5. Mutations within the coding region of CK1ε were identified in several cancers, including ovarian, head and neck and breast cancers 3, 6. Relevant studies have reported that CK1 inhibitors may be drug candidates for treating alcoholism 7.

Gene polymorphisms and environmental factors combined determine individuals' susceptibility to oral cancer 8-13. Identifying genetic factors and their SNPs associated with the development and progression of cancer are challenging tasks and are often used in early detection to determine the most effective treatment options 14, 15. To the best of our knowledge, this study is the first to target the *CK1ε* genetic polymorphism and compare it with environmental factors, such as betel-quid chewing, tobacco use, and alcohol consumption, to determine their effects on oral-cancer susceptibility in Taiwan.

## Materials and Methods

### Subjects and specimen collection

741 oral cancer patients who were recruited at Changhua Christian Hospital in Changhua and Chung Shan Medical University Hospital in Taichung, Taiwan between April, 2007 and April, 2013. Medical information of the oral cancer group, including the American Joint Committee on Cancer (AJCC) clinical stage, the tumor size, lymph node metastasis, distant metastasis and histologic grade, was obtained from their medical records. This study was approved by the Institutional Review Board of Changhua Christian Hospital (CCH No: 161224) and Chung Shan Medical University Hospital (CSMUH No: CS13214-1).

### Selection of casein kinase 1 epsilon Polymorphisms

To obtain adequate power for evaluating the potential association, we investigated rs135745, rs135764, rs1997644 and rs2075984, those with minor allele frequencies greater than 5%. Furthermore, these SNPs of CK1ε gene were selected in this study since these SNPs were associated with the progression of the cancer 16, 17.

### DNA extraction and *CK1ε* genotyping

Genomic DNA was extracted using QIAamp DNA blood mini kits (Qiagen, Valencia, CA, USA) following the manufacturer's instructions as previously described 18, 19. Allelic discrimination assessment for *CK1ε* gene (rs135745, rs135764, rs1997644 and rs2075984) was performed using TaqMan assay with an ABI StepOnePlus™ Software v2.3 real-time PCR system (Applied Biosystems, Foster City, CA, USA).

### Statistical analysis

Multiple logistic regression models were used to analyze the association between *CK1ε* gene (rs135745, rs135764, rs1997644 and rs2075984) genotype frequencies, oral cancer risk and clinical characteristics after controlling for age, betel quid chewing, cigarette smoking, and alcohol consumption. The analyses were performed using the SPSS, and a *p* value less than 0.05 was considered statistically significant.

## Results

Demographical characteristics distribution is presented in Table 1. This study found a significant difference (p < 0.001) in the demographical distributions of age, smoking status, alcohol intake, and betel-quid chewing between the control subjects and oral cancer patients (Table 1).

The frequencies of genetic polymorphisms (rs135745, rs135764, rs1997644 and rs2075984) of the control group were in Hardy-Weinberg equilibrium. The people with the GC variants of the rs135745 polymorphism had 1.41-fold (95% CI: 1.036-1.919; p = 0.029) increased risk of developing oral cancer respectively to their corresponding WT gene (Table 2). However, no significant differences were observed between patients with oral cancer having rs135764, rs1997644 and rs2075984 polymorphisms of the *CK1ε* gene and those with the wild-type (WT) gene.

Tables 3 and 4 demonstrate the associations between environmental risk factors and genetic polymorphisms of *CK1ε*. Among 821 smokers who had *CK1ε* polymorphic rs135745, rs135764, rs1997644, or rs2075984 genes and who had a betel-quid chewing habit had 19.031- (95% CI: 9.840-36.807; p < 0.001), 9.196- (95% CI: 5.250-16.108; p < 0.001), 10.572- (95% CI: 6.001-18.627; p < 0.001) and 9.476-fold (95% CI: 5.383-16.681; p < 0.001) higher risks of developing oral cancer, respectively, compared with individuals with the WT gene (Table 3).

To clarify the role of *CK1ε* genetic polymorphisms in oral cancer clinical statuses, such as tumor size, lymph node or distant metastasis, and histological grade, the distribution frequency of clinical statuses and *CK1ε* genotype frequencies in patients with oral cancer were estimated. As sown in Table 4, we found that patients with the *CK1ε* rs135745 variant genotypes (GC+CC) were significantly associated with lymph-node metastasis (1.622; 95% CI: 1.037-2.536; p = 0.034) (Table 4).

## Discussion

Oral cancer in Taiwan is increasing at an alarming rate compared with other countries, which might be because of the alcohol consumption, tobacco use, and betel-quid chewing habits of the Taiwanese 14, 15, 20. SNPs in genes, which regulate major pathways in the cells, are associated with greater susceptibility to oral cancer 21. In this study, we determined the SNPs in the *CK1ε* gene are associated with clinicopathological statuses of oral cancer. CK1ε is a serine/threonine-specific phosphotransferase that controls major cellular pathways 22. CK1ε expression was correlated with the *c-MYC* oncogene in colon, lung, and breast cancer tumors 23. Varghese et al., reported that CK1ε expression is associated with glioblastoma cell survival 24. Gene expression analysis data showed that CK1ε is overexpressed in several cancer tissue samples compared with in normal tissues. Furthermore, knockdown of CK1ε induces tumor cell-selective cytotoxicity 25.

In our study, a significant difference was found in demographical distributions of age, smoking status, alcohol intake, and betel-quid chewing between control subjects and oral cancer patients, which have also been observed in most relevant studies. We investigated the rs135745, rs1997644, rs2075984, and rs135764 polymorphisms of the *CK1ε* gene. Our results showed that oral cancer patients with the rs135745 polymorphism of *CK1ε* gene increased the risk of developing oral cancer. Hsu's group reported that even in the same cancer, CK1ɛ seems to exhibits diverse functions; thus, it could be an onco-protein or a tumor suppressor 26. We further tested clinicopathological statuses, such as T classification, lymph-node metastasis, distant metastasis, histologic grade, and AJCC cancer stage, and observed significant lymph-node metastases in oral cancer patients with the rs135745-*CK1ε* polymorphic gene. Future studies should investigate the mechanism by which these SNPs modify the clinicopathological status of oral cancer.

Individuals exposed to environmental carcinogens have increased risks of oral cancer 27. Studies have stated that SNPs and oral cancer-associated environmental risk factors have a synergetic effect that can influence oral-cancer development 28. The synergistic effects of environmental factors, betel-quid chewing, smoking, and the four CK1ɛ gene SNPs (rs135745, rs135764, rs1997644 and rs2075984) on the risk of oral cancer were studied. Mutations and changes in the expression and/or activity of CK1 isoforms are often identified in various cancers, such as ovarian and breast cancer as well as adenocarcinoma of the pancreas 6. This study identified the *CK1ε* gene polymorphisms in oral cancer. However, the detailed mechanism of the CK1ɛ genetic polymorphism to oral cancer remained unclear. In the future, some studies are required to elucidate the mechanisms of *CK1ɛ* genetic polymorphisms in oral cancer progression.

In conclusion, our results suggested that the *CK1ɛ*-rs135745 G/C gene polymorphism significantly increased the risk of oral cancer, and furthermore, the combined effects of *CK1ɛ* gene polymorphisms with environmental risk factors increased sensitivity to oral cancer.

## Figures and Tables

**Table 1:** Distributions of demographical characteristics in 462 controls and 741 male patients with oral cancer.

Variable	Controls (*N*=462)	Patients (*N*=741)	*p* value
**Age (yrs)**			
	47.5 ± 13.88	54.5 ± 11.13	*p* <0.001*
**Betel quid chewing**			
No	375 (81.2%)	158 (21.3%)	
Yes	87 (18.8%)	583 (78.7%)	*p* <0.001*
**Cigarette smoking**			
No	275 (59.5%)	107 (14.4%)	
Yes	187 (40.5%)	634 (85.6%)	*p* <0.001*
**Alcohol consumption**			
No	309 (66.9%)	320 (43.2%)	
Yes	153 (33.1%)	421 (56.8%)	*p*<0.001*
**Stage**			
I+II		349 (47.1%)	
III+IV		392 (52.9%)	
**Tumor T status**			
T1+T2		408 (55.1%)	
T3+T4		333 (44.9%)	
**Lymph node status**			
N0		492 (66.4%)	
N1+N2+N3		249 (33.6%)	
**Metastasis**			
M0		732 (98.8%)	
M1		9 (1.2%)	
**Cell differentiation**			
Well differentiated		113 (15.2%)	
Moderately or poorly differentiated		628 (84.8%)	

The Mann-Whitney *U*-test or Fisher's exact test was used between healthy controls and patients with oral cancer. * *p*<0.05.

**Table 2:** The odds ratio (OR), adjusted OR (AOR), and 95% confidence interval (CI) of oral cancer associated with CK1 epsilon genotypic frequencies

Variable	Controls (*N*=462) (%)	Patients (*N*=741) (%)	OR (95% CI)	AOR (95% CI)^a^
**rs135745**				
GG	341 (73.8%)	497 (67.1%)	Reference	Reference
GC	111 (24.0%)	229 (30.9%)	1.39 (1.052-1.834)*	1.41 (1.036-1.919)*
CC	10 (2.2%)	15 (2.0%)	0.95 (0.417-2.171)	0.85 (0.331-2.197)
GC+CC	121 (26.2%)	243 (32.8%)	1.32 (1.010-1.724)*	1.34 (0.992-1.799)
**rs135764**				
GG	331 (71.6%)	532 (71.8%)	Reference	Reference
GA	123 (26.6%)	190 (25.6%)	0.93 (0.679-1.278)	0.98 (0.692-1.400)
AA	8 (1.7%)	19 (2.6%)	1.45 (0.577-3.665)	1.80 (0.671-4.832)
GA+AA	131 (28.3%)	209 (28.2%)	0.94 (0.698-1.274)	1.03 (0.739-1.444)
**rs1997644**				
GG	187 (40.5%)	322 (43.5%)	Reference	Reference
GA	221 (47.8%)	327(44.1%)	0.84 (0.652-1.083)	0.85 (0.640-1.130)
AA	54 (11.7%)	92 (12.4%)	1.02 (0.689-1.520)	0.90 (0.574-1.398)
GA+AA	275 (59.5%)	419 (56.5%)	0.88 (0.691-1.118)	0.87 (0.664-1.137)
**rs2075984**				
AA	159 (34.4%)	246 (33.2%)	Reference	Reference
AC	221 (47.8%)	371 (50.1%)	1.19 (0.880-1.621)	1.13 (0.802-1.580)
CC	82 (17.8%)	124 (16.7%)	0.98 (0.694-1.377)	1.13 (0.676-1.885)
AC+CC	303 (65.6%)	495 (66.8%)	1.17(0.864-1.579)	1.13 (0.805-1.573)

The OR with its 95% CI was estimated by logistic regression models. The AOR with its 95% CI was estimated by multiple logistic regression models after controlling for age, betel quid chewing, cigarette smoking, and alcohol consumption. * *p*<0.05.

**Table 3:** Associations of the combined effect of CK1 epsilon gene polymorphisms and betel nut chewing with the susceptibility to oral cancer among 821 smokers.

Variable	Controls (N=187) (%)	Patients (N=634) (%)	OR (95% CI)	AOR (95% CI)
**rs135745**				
GG genotype and non betel-nut chewing	87 (46.5%)	58 (9.1%)	Reference	Reference
GC or CC genotype or betel-nut chewing	87 (46.5%)	393 (62.0%)	6.776 (4.518-10.163)	6.150 (4.069-9.296)
GC or CC genotype with betel-nut chewing	13 (7.0%)	183 (28.9%)	21.115 (10.986-40.583)	19.031 (9.840-36.807)
				
**rs135764**				
GG genotype and non betel-nut chewing	84 (44.9%)	60 (9.5%)	Reference	Reference
GA or AA genotype or betel-nut chewing	80 (42.8%)	422 (66.6%)	7.385 (4.908-11.111)	6.988 (4.597-10.621)
GA or AA genotype with betel-nut chewing	23 (12.3%)	152 (24.0%)	9.252 (5.340-16.030)	9.196 (5.250-16.108)
				
**rs1997644**				
GG genotype and non betel-nut chewing	47 (25.1%)	31 (4.9%)	Reference	Reference
GA or AA genotype or betel-nut chewing	96 (51.3%)	287 (45.3%)	4.533 (2.725-7.540)	4.366 (2.586-7.369)
GA or AA genotype with betel-nut chewing	44 (23.5%)	316 (49.8%)	10889 (6.267-18.917)	10.572 (6.001-18.627)
				
**rs2075984**				
AA genotype and non betel-nut chewing	42 (22.5%)	32 (5.0%)	Reference	Reference
AC or CC genotype or betel-nut chewing	99 (52.9%)	238 (37.5%)	3.155 (0.1883-5.287)	2.990 (1.759-5.080)
AC or CC genotype with betel-nut chewing	46 (24.6%)	364 (57.4%)	10.386 (5.975-18.052)	9.476 (5.383-16.681)

The adjusted odds ratio (OR; AOR) with its 95% confidence interval (CI) was estimated by multiple logistic regression models after controlling for age and alcohol consumption.

**Table 4:** Distribution frequency of clinical status and CK1 epsilon rs135745 genotype frequencies in 741 patients with oral cancer.

Variable	GG (N = 497) *n* (%)	GC + CC (N = 244) *n* (%)	OR (95% CI)	AOR (95% CI)
**Stage**				
I/II	238 (47.9%)	111 (45.5%)	Reference	Reference
III//IV	259 (52.1%)	133 (54.5%)	0.795 (0.480-1.629)	0.796 (0.481-1.319)
**T classification**				
T1/T2	274 (55.1%)	134 (54.9%)	Reference	Reference
T3/T4	223 (44.9%)	110 (45.1%)	1.082 (0.732-1.600)	1.081 (0.731-1.598)
**Lymph node metastasis**				
No	342 (68.8%)	150 (61.5%)	Reference	Reference
Yes	155 (31.2%)	94 (38.5%)	1.623 (1.038-2.537)*	1.622 (1.037-2.536)*
**Distant metastasis**				
No	490 (98.6%)	242 (99.2%)	Reference	Reference
Yes	7 (1.4%)	2 (0.8%)	0.458 (0.093-2.259)	0.461 (0.093-2.277)
**Histological grade**				
Well	78 (15.7%)	35 (14.3%)	Reference	Reference
Moderate/Poor	419 (84.3%)	209 (85.7%)	1.052(0.679-1.629)	1.055 (0.995-1.007)

The OR with its 95% CI was estimated by logistic regression models. The AOR with its 95% CI was estimated by multiple logistic regression models after controlling for age, betel quid chewing, cigarette smoking, and alcohol consumption. * *p*<0.05.
